# Veratrum Viride as a Poison and as a Remedial Agent

**Published:** 1857-11

**Authors:** J. Stayton Pashley

**Affiliations:** Waupaca Co., Wisconsin


ARTICLE III.—*Veratrum Viride as a poison and as a remedial agent.* By J. Stayton Pashley, Waupaca Co., Wisconsin.

Dear Sir,—Recognizing the fact that the Veratrum Viride or American Hellebore is being more generally used than formerly by the medical practitioner in the North-West, I am induced to publish the minutes of a case of interest to those of our profession who were present, and in which the life of one of our best citizens was at stake.

In the month of March last, Mr. C. O. B——, of good constitution and in ordinary health, entered our office, and complaining of slight indisposition, passed to the rear end of the room where we usually kept alcohol and other liquors, of which fact he was cognizant, and taking up a tincture bottle which was *not labelled*, and into which we had just filtered from a *labelled* bottle *a saturated tincture of Veratrum Viride*, he smelled of it hastily, and supposing it to be brandy, poured into a tumbler about ℥ij (as we afterwards ascertained) and quaffed the delicious draught!

The impression made upon his gustatory nerves was far from agreeable.

He walked, or rather *staggered* forward, about fifteen or twenty feet, when he fell to the floor, and commenced vomiting violently and complaining of distressing dyspnœa and total blindness. His appearance at this juncture was striking—surface blanched and covered with a profuse cold sweat, eyes sunken and fixed, pupil of the eye dilated, a perfect Hippocratic face; body flexed, and a strong disposition to muscular contraction or rigidity; pulse falling rapidly, until it was imperceptible at the wrist, and the heart's action was quite feeble.

Purging early supervened on the vomiting, and both persisted until the remedies prescribed produced their effect. Fortunately my partner, Dr. J. E. Thayer, was at hand to render assistance, and the patient was subjected to the following treatment:

3 p. m. (time of accident.)—Gave of alcohol (98 per cent.) ℥ij, and tinct. opii ʒi, with water.

3.30 p. m.—No perceptible effect. Gave sulphate of morphine grj, and brandy ℥j.

4 p. m.—Vomiting and purging less frequent, but excessive straining; pulse scarcely perceptible. Gave of morphine, grss., with brandy and the white of eggs.

4.30 p. m.—Purging entirely ceased; vomiting nearly so; pulse rising. Gave morphine, gr. $\frac{1}{4}$, with brandy and albumen as before, and applied blister over epigastrum.

5 p. m.—Patient is perfectly quiet, and disposed to sleep; pulse nearly normal, yet a little soft.

During the evening, and nearly all night, the morphine and brandy in small doses were administered, and in the morning the patient was conveyed to his residence, but for some days complained of tenderness over the stomach and small intestines. A proper regimen was prescribed, and our friend still lives, cautioning others to "Touch not, taste not, nor handle not," anything pertaining to a physician's office.

As a remedial agent, I have found the Veratrum to be almost invaluable in any case where one of the indications is to reduce the force of the circulation, provided there is not already too great depression of the vital forces.

Norwood's preparation not being always attainable, I have used a *saturated* tincture of my own preparation, and always in combination with an opiate and diuretic.

The most usual formula is the following:

| | | |
|---|---|---|
| ℞ | Tinct. Veratrum | ʒi |
| | " Opii Camph. | |
| | Sp. Nit. Dulc. *aa.* | ℥j |

Dose for an adult: One teaspoonful—to be increased or diminished as the case may demand.

My object in adding the opium is to control the tendency which this agent (as well as tartarized antimony and ipecac.) has to develope intestinal irritation. The diuretic is introduced to prevent the arrest of the secretion from the kidneys, which opium is liable to effect.

